# Supplementary material for: Efficient De Novo Biosynthesis of Heme by Membrane Engineering in Escherichia coli
Source: Int J Mol Sci. 2022 Dec 8;23(24):15524. doi: 10.3390/ijms232415524 (PMC9779679; doi:10.3390/ijms232415524)

SUPPLEMENTARY MATERIAL

Supplementary Table S1. Strains and plasmids used in this study.

Strains or plasmids	Relevant genotype/property	Origin
**Strains**		
E. coli DH5α	*recA1 supE44 endA1 hsdR17 gyrA96 relA1 thi-1 Δ*(*lac-proAB*)*/F ′* [*traD36proAB+lacIq E. coli lacZΔM15*]	Lab stock
E. coli JM109	*F-φ80 dlacZ ΔM15 Δ*(*lacZYA-argF*)*U169 endA1 recA1 hsdR17* (*rK- mK+*) *deoR thi-1 phoA supE44λ- gyrA96 relA1*	Lab stock
E. coli BL21(DE3)	F^- *ompT* $hsdS_B(r_B^- m_B^-)$ *gal dcmrne131* λ(DE3)	Novagen
E. coli JM109(DE3)	*endA1 glnV44 thi-1 relA1 gyrA96 recA1* $mcrB^+$ Δ(*lac-proAB*) e14- [F' *traD36* $proAB^+$ *lacIq lacZ*ΔM15] $hsdR17(r_K^- m_K^+)$ λ(DE3)	Promega
E. coli NovaBlue(DE3)	*endA1 hsdR17* $r_{k12}^- m_{k12}^+$ *supE44 thi-1 relA1 gyrA96 recA1 lac* F'[$proA^+B^+ lacI^q$ *lacZ*ΔM15: Tn*10*] (Tet^R) λ(DE3)	Novagen
E. coli Turner(DE3)	F^- *ompT* $hsdS_B$ $(r_B^- m_B^-)$ *gal dcm lacY1*	Novagen

	λ(DE3)	
EBL-pET-A	*E. coli* BL21(DE3) contains pET-*hemA*	This study
EBL-pET-AL	*E. coli* BL21(DE3) contains pET-*hemA*-*hemL*	This study
EBL-pCD-A	*E. coli* BL21(DE3) contains pCD-*hemA*	This study
EBL-pCD-AL	*E. coli* BL21(DE3) contains pCD-*hemA*-*hemL*	This study
EBL-pAC-A	*E. coli* BL21(DE3) contains pAC-*hemA*	This study
EBL-pAC-L	*E. coli* BL21(DE3) contains pAC-*hemL*	This study
EBL-pAC-AL	*E. coli* BL21(DE3) contains pAC-*hemA*-*hemL*	This study
EJM-pET-A	*E. coli* JM109(DE3) contains pET-*hemA*	This study
EJM-pET-AL	*E. coli* JM109(DE3) contains pET-*hemA*-*hemL*	This study
EJM-pCD-A	*E. coli* JM109(DE3) contains pCD-*hemA*	This study
EJM-pCD-AL	*E. coli* JM109(DE3) contains pCD-*hemA*-*hemL*	This study
EJM-pAC-A	*E. coli* JM109(DE3) contains pAC-*hemA*	This study
EJM-pAC-L	*E. coli* JM109(DE3) contains pAC-*hemL*	This study
EJM-pAC-AL	*E. coli* JM109(DE3) contains pET-*hemA*-*hemL*	This study

ENO-pET-A	*E. coli* NovaBlue(DE3) contains pET-*hemA*	This study
ENO-pET-AL	*E. coli* NovaBlue(DE3) contains pET-*hemA*-*hemL*	This study
ENO-pCD-A	*E. coli* NovaBlue(DE3) contains pCD-*hemA*	This study
ENO-pCD-AL	*E. coli* NovaBlue(DE3) contains pCD-*hemA*-*hemL*	This study
ENO-pAC-A	*E. coli* NovaBlue(DE3) contains pAC-*hemA*	This study
ENO-pAC-L	*E. coli* NovaBlue(DE3) contains pAC-*hemL*	This study
ENO-pAC-AL	*E. coli* NovaBlue(DE3) contains pAC-*hemA*-*hemL*	This study
ETU-pET-A	*E. coli* Turner(DE3) contains pET-*hemA*	This study
ETU-pET-AL	*E. coli* Turner(DE3) contains pET-*hemA*-*hemL*	This study
ETU-pCD-A	*E. coli* Turner(DE3) contains pCD-*hemA*	This study
ETU-pCD-AL	*E. coli* Turner(DE3) contains pCD-*hemA*-*hemL*	This study
ETU-pAC-A	*E. coli* Turner(DE3) contains pAC-*hemA*	This study

ETU-pAC-L	*E. coli* Turner(DE3) contains pAC-*hemL*	This study
ETU-pAC-AL	*E. coli* Turner(DE3) contains pAC-*hemA*-*hemL*	This study
EJM-Dpp-△CyoE-pCD-AL	Recombinant strains overexpressing the Dpp pathway by gene editing after knockout of gene CyoE	This study
EJM-Ccm-△CyoE-pCD-AL	Recombinant strains overexpressing the Ccm pathway by gene editing after knockout of gene CyoE	This study
EJM-△CysG-pCD-AL	Recombinant strain after knockout of gene CysG	This study
EJM-△hemX-pCD-AL	Recombinant strain after knockout of gene hemX	This study
EJM-△CyoE-pCD-AL	Recombinant strain after knockout of gene CyoE	This study
**Plasmids**		
pETDuet-1	Double T7 promoters; ColE1 ori AmpR	Novagen
pCDFDuet-1	Double T7 promoters; CloDF13 ori StrR	Novagen
pACYCDuet-1	Double T7 promoters; P15A ori CmR	Novagen
pKD13	Helper plasmids for Red/ET homologous recombination	Lab stock

pKD46	Helper plasmids for Red/ET homologous recombination	Lab stock
pET-*hemA*	pETDuet-1 derivate	This study
pET-*hemL*	pETDuet-1 derivate	This study
pCD-*hemA*	pCDFDuet-1 derivate	This study
pCD-*hemL*	pCDFDuet-1 derivate	This study
pAC-*hemA*	pACYCDuet-1 derivate	This study
pAC-*hemL*	pACYCDuet-1 derivate	This study
pET-*hemA-hemL*	pETDuet-1 derivate	This study
pCD-*hemA-hemL*	pCDFDuet-1 derivate	This study
pAC-*hemA-hemL*	pACYCDuet-1 derivate	This study

Supplementary Table S2. The primers used in this study.

**Primers**	**Sequences（5′→3′）**
hemA-F	ATACATATGGCAGATATGACCCTTTTAGCACTCGGTATC
hemA-R	GGGTACCGACGTCAGCTACTCCAGCCCGAGGC
hemL-F	TAATAAGGAGATATACCATGAGTAAGTCTGAAAATCTTTACAGCGC
hemL-R	CTGTGGTGATGATGGTGATGCAACTTCGCAAACACCCGA
pACZT-F	CTGACGTCGGTACCCTCG
pACZT-R	ATCTGCCATATGTATATCTCCTTCTTATACTTAAC

pCDZT-F	CTGACGTCGGTACCCTCG
pCDZT-R	ATCTGCCATATGTATATCTCCTTCTTATACTTAAC
pETZT-F	CTGACGTCGGTACCCTCG
pETZT-R	ATCTGCCATATGTATATCTCCTTCTTATACTTAAC
pACYZA-F	TTGTACACGGCCGCATAATC
pACYZA-R	CGCTGGCCTTCATATGACC
pACKCZ-F	CATCACCATCATCACCACAGC
pACKCZ-R	GGTATATCTCCTTATTAAAGTTAAACAAAATTATTTCTACAG
pACYZAL-F	TCCGCTGGTCGTTAACCAC
pACYZAL-R	GCTAGTTATTGCTCAGCGG
pCDYZA-F	TTGTACACGGCCGCATAATC
pCDYZA-R	CGCTGGCCTTCATATGACC
pCDKCZ-F	CATCACCATCATCACCACAGC
pCDKCZ-R	GGTATATCTCCTTATTAAAGTTAAACAAAATTATTTCTACAG
pCDYZAL-F	TCCGCTGGTCGTTAACCAC
pCDYZAL-R	GCTAGTTATTGCTCAGCGG
pETYZA-F	TTGTACACGGCCGCATAATC
pETYZA-R	CGCTGGCCTTCATATGACC
pETKCZ-F	CATCACCATCATCACCACAGC
pETKCZ-R	GGTATATCTCCTTCTTAAAGTTAAACAAAATTATTTCTAG
pETYZAL-F	TCCGCTGGTCGTTAACCAC
pETYZAL-R	GCTAGTTATTGCTCAGCGG

kanR-F	GGCTGGAGCTGCTTCGAAG
kanR-R	CGACCTGCAGTTCGAAGTTCC
hemX-up-F	GGCCCCGACTACCTGATTATTTCGCC
hemX-up-R	CTTCGAAGCAGCTCCAGCCCGGAGGTTTTTCTTGTTCCG
hemX-down-F	GGAACTTCGAACTGCAGGTCGTGATACTCCGGCAGC
hemX-down-R	GCAGCCAGTAGATAGTTCACCACCG
cyoE-up-F	CAGCGTTCTTTGCGCTGGTCGG
cyoE-up-R	CTTCGAAGCAGCTCCAGCCTCGTTACTTGCAGGTATTGCTTAA ACATC
cyoE-down-F	GGAACTTCGAACTGCAGGTCGTATGGTACCGGACTCGCATAC
cyoE-down-R	GCCAGCCCACCGACAATTAATGG
cysG-up-F	GTGGGCAATCCTGCCGCAAACC
cysG-up-R	CTTCGAAGCAGCTCCAGCCATTGGCAAAATATAGGCAAATGAT CCAC
cysG-down-F	GGAACTTCGAACTGCAGGTCGGCGATAAACTGAACTGGTTCT CC
cysG-down-R	CTGTCAGAGACTTACTGTGCAGTAGG
kanF-YZ	CTTGTCCAGATAGCCCAGTAGC
dppA-up-F	GCGTTACTTATGGCTGGCCCTG
dppA-up-R	CTTCGAAGCAGCTCCAGCCTATTCTGCTCCAATTGTGATGTTT GTTG
dppA-T7-F	GGAACTTCGAACTGCAGGTCGCTCGATCCCGCGAAATTAATAC

	G
dppA-T7-R	GCTGCTGCCCATGGTATATC
dppA-F	GATATACCATGGGCAGCAGCATGCGTATTTCCTTGAAAAAGTCAGG
dppA-R	CGAAGTATTCGTAGCTGCCGCCAG
ccmA-up-F	CCAATACCGAAGAGTTCTGCATTAGCTGCC
ccmA-up-R	CTTCGAAGCAGCTCCAGCCGCAATAACCCTGTTAAAAACCTGGCTC
ccmA-T7-F	GGAACTTCGAACTGCAGGTCGCTCGATCCCGCGAAATTAATACG
ccmA-T7-R	GCTGCTGCCCATGGTATATC
ccmA-F	GGAGATATACCATGGGCAGCAGCGTGGGTATGCTTGAAGCCAGAGAG
ccmA-R	GCTCGTCGAGGATCCATAACGTGG

Supplementary Table S3. This table shows the ALA and heme production detected by fermentation of recombinant strains constructed in this study.

Strain	Characterization	ALA (mg/L)	Heme (mg/L)
EBL-pAC-AL	E. coli BL21(DE3) contains pAC-hemA-hemL	49.04 ± 0.59	not detected
EJM-pAC-AL	E. coli JM109(DE3) contains pET-hemA-hemL	73.2 ± 1.42	not detected
EBL-pET-AL	E. coli BL21(DE3) contains pET-hemA-hemL	24.78 ± 0.5	not detected
EJM-pET-AL	E. coli JM109(DE3) contains pET-hemA-hemL	82.62 ± 0.34	not detected

Supplementary Figure S1. Growth curve and ALA production of recombinant strain fermented for 72 h.

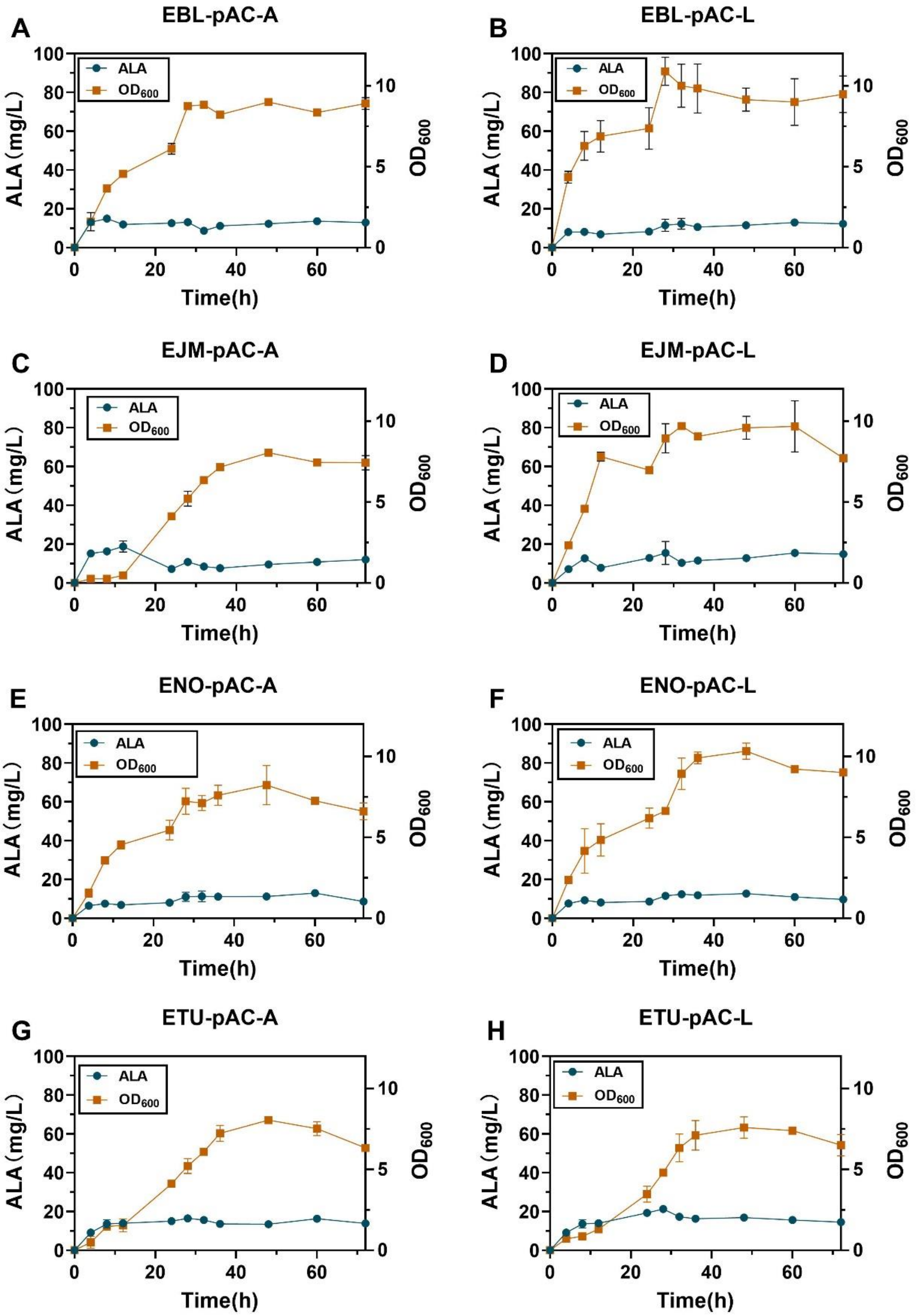

Supplementary Figure S2. HPLC chromatograms of heme. (A) the chromatograms obtained from standard heme (20.0 mg/L); (B) the chromatograms obtained from sample.

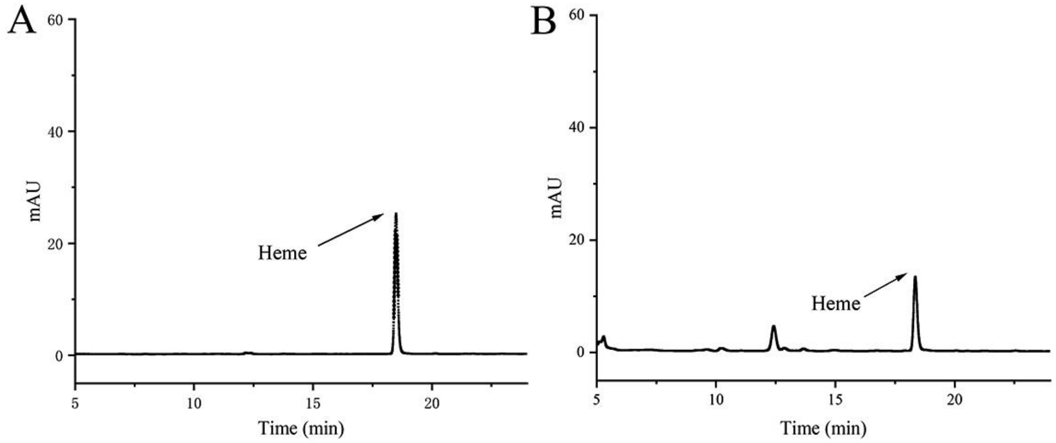

Supplement: Supplementary file 1 [file ijms-23-15524-s001.zip › ijms-2021322-supplementary.pdf]
